# Cytogenetic Study and Pollen Viability of *Phalaenopsis* Queen Beer ‘Mantefon’

**DOI:** 10.3390/plants12152828

**Published:** 2023-07-31

**Authors:** Samantha Serafin Sevilleno, Hye Ryun An, Raisa Aone M. Cabahug-Braza, Yun-Jae Ahn, Yoon-Jung Hwang

**Affiliations:** 1 Department of Convergence Science, Sahmyook University, Seoul 01795, Republic of Korea; samanthasevilleno20@gmail.com; 2 Floriculture Research Division, National Institute of Horticultural & Herbal Science, Rural Development Administration, Wanju 55365, Republic of Korea; hryun@korea.kr; 3 Plant Genetics and Breeding Institute, Sahmyook University, Seoul 01795, Republic of Korea; raisaaone@gmail.com; 4 Department of Horticultural Science, Kyungpook National University, Daegu 41566, Republic of Korea; yjahn0121@gmail.com

**Keywords:** cytogenetics, FISH, micronucleus, orchid, pollen staining, sporads

## Abstract

Intergeneric and interspecific hybridization has been employed for the breeding of *Phalaenopsis* to transfer desirable traits between species, producing novel phenotypes with improved size, color, form, and flower-bearing ability. These characteristics are often enhanced; however, many of these hybrids are triploids and have reduced or complete sterility, for example, *Phalaenopsis* Queen Beer ‘Mantefon’, an important novelty-type cultivar in Asia, particularly in China, Japan, and Republic of Korea. Despite the increasing demand for the crop for ornamental purposes, little is known about its cytogenetics, which is essential for breeding and, consequently, crop improvement. In this study, karyotyping using fluorescence in situ hybridization, meiotic chromosome behavior analysis, pollen staining, and in vitro viability germination tests were performed to understand the cause of hybrid sterility and pollen abnormality in *Phalaenopsis* Queen Beer ‘Mantefon’ from a cytogenetic perspective. Viability tests revealed pollen infertility at all flower developmental stages, confirmed by the absence of pollen tube growth. Aberrant chromosomal behavior was observed in pollen mother cells (PMCs), frequently forming univalents, chromosomal bridges, and laggards during the entire meiotic process. PMCs were also divided irregularly into sporads with varying numbers of micronuclei, which may be responsible for pollen sterility in this cultivar. Altogether, the cytogenetic analyses provided insights into the pollen development of *Phalaenopsis* Queen Beer ‘Mantefon’ and the conceivable causes of its infertility.

## 1. Introduction

*Phalaenopsis*, also called moth orchids, are among the most popular and economically important potted plants and cut flowers in the ornamental market due to their ease of cultivation under artificial conditions [1,2]. Moreover, *Phalaenopsis* hybrids can be scheduled to flower without difficulty throughout the year and have attractive and long-lasting inflorescences that usually last up to 4 months [3]. In 2020, the total wholesale value of potted orchids, including *Phalaenopsis*, reached USD 276 million [4].

Over the past century, global demand for new *Phalaenopsis* cultivars has increased. One essential novelty-type cultivar in Asia, particularly in China, Japan, and Republic of Korea, is the *Phalaenopsis* Queen Beer ‘Mantefon’ owing to its strong red-/purple-colored flowers and multiple small flowers in one flower spike [5]. This cultivar possesses triploid (2*n* = 3x = 57) chromosomes of various sizes (small, medium, and large). Despite being a novel cultivar with desirable morphological characteristics, it has impaired fertility; therefore, it is disadvantageous to use for hybridization [6]. Breeding programs for orchids often rely on interspecific hybridization using both wild species and commercial cultivars to combine traits, such as the colors, multiflorous state, and heavier and larger flowers, to improve their horticultural value and quality [7]. Unfortunately, the process of assessing hybrids is slow as it takes up to 6 years to obtain a blooming F_2_ hybrid [8]. Similar to diploid species (2*n* = 2x = 38), these hybrids often feature variations in chromosomal characteristics [9]. Crossing two species with different chromosome numbers or sizes usually results in reduced or complete sterility in progenies, such as in the case of *P.* Queen Beer ‘Mantefon’, which can be attributed to abnormalities in the patterns of segregation of chromosomes during meiosis [6,10].

Meiosis is a key feature of sexual reproduction in which four haploid gametes are generated from diploid progenitor cells. The first meiotic division involves chromosome pairing, recombination, and segregation, whereas sister chromatids are separated during the second meiotic division [11]. The four haploid cells, called tetrads, undergo two rounds of mitosis and develop into mature pollen [12]. Meiotic recombination and random segregation of homologous chromosomes combine to create genetic variation [13,14]. The behavior of chromosomes during meiosis significantly affects plant fertility. Successful meiosis, which entails regular chromosome pairing and segregation, results in the production of fertile pollen [15]. However, abnormal interactions between non-homologous chromosomes may have detrimental effects, such as impaired pollen viability [11]. Unpaired univalents, homoeologous bivalents, or multivalents caused by aberrant chromosome pairing can cause the missegregation of meiotic chromosomes, resulting in reduced fertility [16,17]. These meiotic irregularities are commonly observed in interspecific or intergeneric hybrids of *Aranda* [18], *Dendrobium* Lindl. [19], *Doritaenopsis* [20], and *Paphiopedilum* Pfitz [21].

Chromosome pairing analysis in *Phalaenopsis* is challenging since (1) plants grow slowly and require approximately 2 to 3 years to reach maturity; (2) each plant produces very few flowers, hindering the collection of sufficient microsporocytes at the right stages for analysis; (3) microsporocytes are enclosed in a thick callose wall, hampering stain penetration; and (4) meiotic chromosomes cannot spread well due to clumping and stickiness [8]. These obstacles may be overcome by using fluorescence in situ hybridization (FISH), a revolutionary cytogenetic technique that generally involves the use of genomic DNA or a portion of genomic DNA as a probe [22]. Conserved repetitive sequences in 45S and 5S rDNAs are widely used to analyze their evolutionary origins and identify chromosomes and ploidy levels [23]. Genomic probes are widely used to discriminate chromosomes from two or more allopolyploid species and distinguish the formation and evolution of different sources of polyploid species arising from chromosomal translocation and loss, gene insertion, or chromosome-derived changes [24,25,26,27].

Basic data, such as chromosome composition, meiotic behavior, and pollen fertility, are essential for determining the genetic variability of a species, which aids in germplasm characterization, biodiversity studies, and the selection of plants to be included in plant breeding programs [28,29]. Therefore, in this study, we aimed to analyze the chromosome composition and behavior during the process of meiosis, as well as the pollen viability of the *P.* Queen Beer ‘Mantefon’ cultivar through staining and in vitro germination tests.

## 2. Results

### 2.1. FISH Karyotype

**Figure 1:**
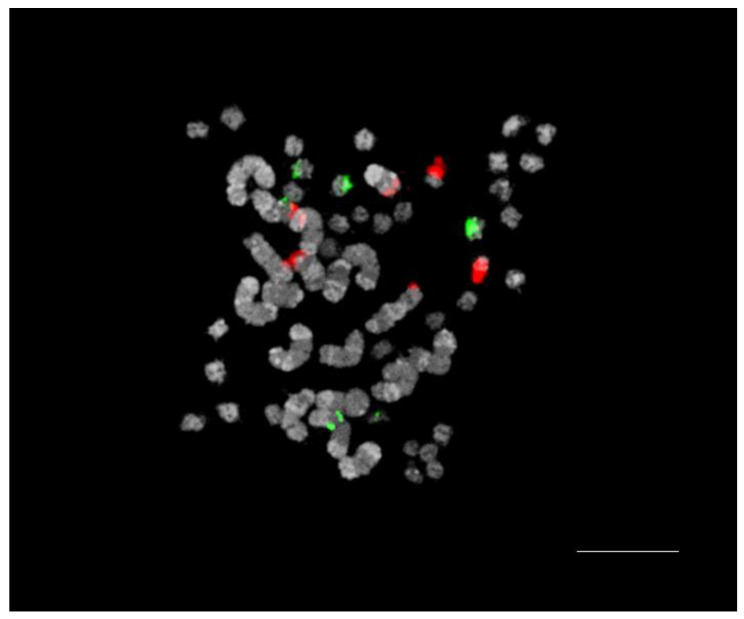
FISH using 5S rDNA (green fluorescence) and 45S rDNA (red fluorescence) as probes in the somatic metaphase chromosomes of *Phalaenopsis* Queen Beer ‘Mantefon’. Scale bar = 10 μm.

*P.* Queen Beer ‘Mantefon’ was confirmed as a triploid with 2*n* = 3x = 57 asymmetrical chromosomes, of which 38 were small and 19 were long (Figure 1). Six 5S rDNA loci were found on two sets of chromosomes. A single locus of 5S rDNA was observed on one large chromosome, whereas five other loci were localized on small chromosomes. However, the same number of 45S rDNA loci was found, in which the two pairs were localized on the large chromosomes and a single pair on the small chromosomes.

### 2.2. Pollen Viability Evaluation by Staining and In Vitro Germination

**Figure 2:**
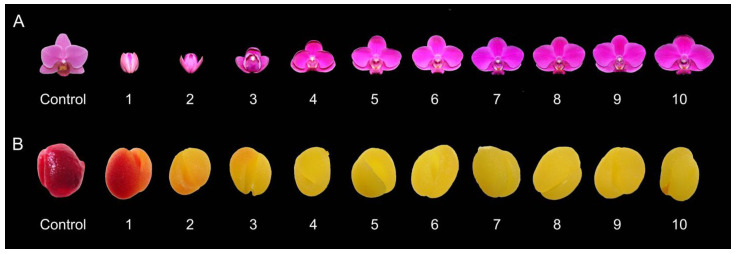
Flower developmental stages (**A**) and their corresponding pollen viability evaluated by TTC staining (**B**) in *Phalaenopsis* Queen Beer ‘Mantefon’. (**A**) Flower developmental stages are defined as (1) tight flower bud, (2) sepal half-open, (3) flower half-open, (4) prior to anthesis, (5) flower fully open, (6) one day after fully open, (7) two days after fully open, (8) three days after fully open, (9) four days after fully open, and (10) five days after fully open. (**B**) The pollen viability of *Phalaenopsis* Queen Beer ‘Mantefon’ was evaluated by staining with TTC solution. Areas of pollinia stained with red were considered viable, whereas unstained pollinia was considered non-viable. The anthesis stage of *Phalaenopsis* ‘KS Little Gem’ was used as control.

To confirm the sterility of the triploid ‘Mantefon’ cultivar, the pollinia were stained with 2,3,5-triphenyl tetrazolium chloride (TTC) solution to determine their viability. The tetraploid cultivar, *Phalaenopsis* ‘KS Little Gem’ in the anthesis stage was used as control. The results showed that all freshly collected pollinia from the 10 different flower stages of *P.* Queen Beer ‘Mantefon’ (Figure 2A) remained yellow or unstained (Figure 2B), indicating that the pollinia were non-viable or had low viability, while the control showed prominent viability as revealed by heavy TTC staining.

**Figure 3:**
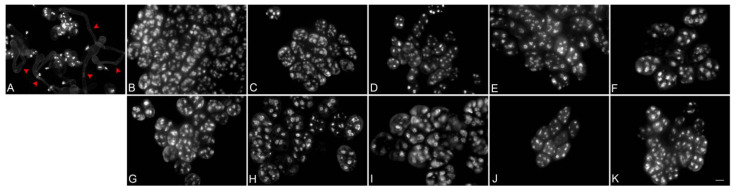
Evaluation of pollen viability in the 10 flower developmental stages of *Phalaenopsis* Queen Beer ‘Mantefon’ using in vitro pollen germination subsequently stained with DAPI. The anthesis stage of *Phalaenopsis* ‘KS Little Gem’ was used as (**A**) control. Red arrows indicate pollen tube growth. The flower stages are as follows: (**B**) Tight flower bud, (**C**) sepal half-open, (**D**) flower half-open, (**E**) prior to anthesis, (**F**) flower fully open, (**G**) one day after fully open, (**H**) two days after fully open, (**I**) three days after fully open, (**J**) four days after fully open, and (**K**) five days after fully open. Pollen failed to grow pollen tubes in all stages. Scale bar = 10 μm.

In vitro germination was conducted to evaluate pollen viability. After 10 days of incubation, pollen tubes were observed in *P.* ‘KS Little Gem’ (Figure 3A) via 1 µg mL^−1^ 4′,6-diamidino-2-phenylindole (DAPI) staining; however, no pollen tube growth was detected at any stage of *P.* Queen Beer ‘Mantefon’ (Figure 3B–K). The results of staining and germination tests confirmed the pollen infertility in this cultivar.

### 2.3. Meiotic Chromosome Behavior of P. Queen Beer ‘Mantefon’

To clarify the reason for the sterility of this cultivar, meiotic chromosome behavior at different stages of pollen mother cells (PMCs) was examined.

**Figure 4:**
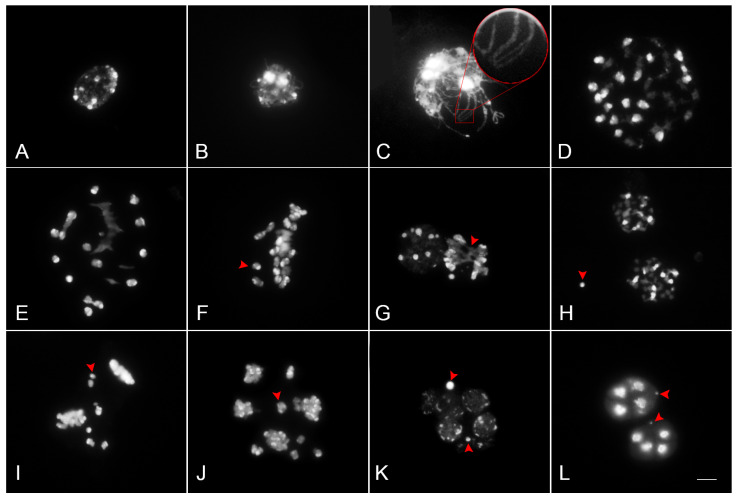
Meiotic chromosome behaviors in PMCs of *Phalaenopsis* Queen Beer ‘Mantefon’. The chromosomes were stained with DAPI. Chromosomes were condensed at leptotene (**A**), and thin threads were observed at zygotene (**B**). Unpaired chromosomes (arrow) were observed in pachytene (**C**), and several univalents (arrow) and multivalents were found in diakinesis to metaphase I (**D**–**F**). At metaphase I, abnormal chromosome disposition was detected, and some chromosomes migrated precociously to the opposite poles (**E**,**F**). Abnormal anaphase with chromosome bridges (arrow) and lagging chromosomes was detected (**G**). Micronucleus was found in telophase I (**H**). Abnormal metaphase II and anaphase II were shown to have lagging chromosomes (arrow) (**I**,**J**). Micronuclei were found in both telophase II (**K**) and the resulting tetrad (**L**). Scale bar = 10 μm.

In prophase I, chromosomes began to condense as long thin threads in leptotene (Figure 4A), and the pairing of homoeologous chromosomes occurred in the zygotene (Figure 4B). Partially synapsed and unpaired homoeologous chromosomes were observed at the pachytene stage (Figure 4C). Chromosome pairing was examined during diakinesis (Figure 4D) until early metaphase I (Figure 4E), showing irregularities in the presence of univalents (I), bivalents (II), and trivalents (III). The average meiotic configuration was 7I + 20.3II + 1.3III (Table 1).

The univalents ranged from 1 to 19, indicating partial failure of uniparental chromosomal pairing and high genomic heterozygosity of ‘Mantefon’. The trivalents ranged from 1 to 4, implying some degree of homology between the two parents of ‘Mantefon’.

In metaphase I, some bivalents were aligned along the equator; however, most of the chromosomes were disorderly aligned on the equatorial plate (Figure 4F). Most anaphase I cells exhibited lagging chromosomes and chromosome bridges (Figure 4G). These lagging chromosomes formed micronuclei, as observed during telophase I (Figure 4H). In the second meiotic division, chromosome bridges and lagging chromosomes were observed (Figure 4I–K). The chromosomes did not segregate synchronously in anaphase I (Figure 4J), resulting in the formation of micronuclei and various meiotic products with different numbers of chromosomes, as detected in telophase II (Figure 4K) and the resulting tetrad (Figure 4L). Throughout meiosis, abnormal chromosome behaviors were observed in most PMCs of *P.* Queen Beer ‘Mantefon’ (Table 2).

### 2.4. Sporad Quantification in PMCs

**Figure 5:**
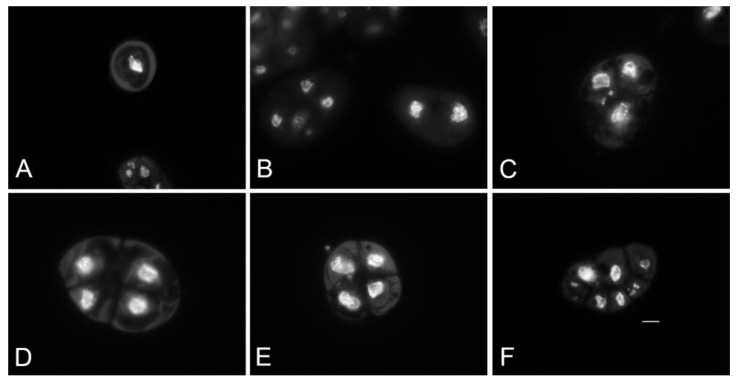
Meiotic products of *Phalaenopsis* Queen Beer ‘Mantefon’. Monad (**A**), dyad (**B**), triad with micronuclei (**C**), normal tetrad (**D**), tetrad with micronucleus (**E**), and polyad with micronuclei (**F**). Scale bar = 10 μm.

Most PMCs were irregularly divided into sporads with varying numbers of micronuclei (Figure 5). Among the meiotic products, 2% monads, 2% dyads, 6% triads, and 11% polyads were recorded. Tetrads with micronuclei were more prominent (44%) than normal tetrads (21%). Sporads with micronuclei comprised 15% of the observed PMCs (Table 3).

## 3. Discussion

Intergeneric and interspecific hybridization has been utilized in orchid breeding to transfer desirable traits between species, producing novel phenotypes with desirable sizes, colors, forms, and floriferousness [23]. In many instances, these characteristics are improved; however, many of these hybrids are triploid and have reduced or complete sterility. The fertility of triploid orchid hybrids differs significantly and affects breeding methods [30].

In a study conducted by Lee et al. [6], triploid cultivars with asymmetrical chromosomes were observed to be infertile or have low fertility, such as those of *P.* Golden Sands ‘Canary’, *P.* Taipei Gold ‘STM’, *P.* Joy Spring Canary ‘Taipei’, *P.* Sogo Relax ‘Sogo F-987’, *P.* Liu’s Berry ‘SW’, and *P.* Queen Beer ‘Mantefon’. The mitotic analysis conducted in this study confirmed that *P.* Queen Beer ‘Mantefon’ is a triploid (2*n* = 3x = 57) possessing chromosomes of different sizes (small to large). Karyotype analysis using conventional staining methods provides basic information, such as chromosome number, size, type, and nucleolus-organizing region of the genome [31]. Although the chromosome number of ‘Mantefon’ has already been investigated in a previous study, to date, no detailed karyotype information essential for genome sequencing research and the development and breeding of this cultivar is available. Molecular cytogenetic techniques, such as FISH, used to identify repetitive sequence families and their distribution in plant chromosomes, have proven to be powerful tools for chromosome characterization [32,33,34]. To the best of our knowledge, this is the first FISH karyotyping report in *P.* Queen Beer ‘Mantefon’ using 18S-5.8S-28S and 5S rDNA sequences as probes.

Pollen forms various types of aggregated entities called pollen dispersal units (PDUs) [35]. In flowering plants, Orchidaceae has the greatest number of PDU types and pollen is often packed in PDUs called pollinia [36,37]. Pollen quality is assessed based on the viability and vigor of the pollen grains. Pollen viability tests are usually used to screen pollen fertility and, primarily, to make the crossing between economically important genotypes safer [38]. Pollen vigor is described as the speed of pollen grain germination and the rate of pollen tube growth [39]. In vitro pollen germination tests have been used to evaluate the percentage of pollen germination and can also be used to assess pollen vigor by monitoring the rate of germination over a specific time or length of pollen tubes [40]. Pollen that fails to germinate typically exhibits poor pollen tube growth, which can result in unsuccessful fertilization [41].

Viability tests using staining solutions, such as TTC [42,43,44,45], Alexander’s dye [46], fluorescein diacetate (FDA) [47], fluorochromatic dye (FCR) [48], lactophenol cotton blue (LPCB) [49], or thiazolyl blue (MTT) [43], are faster and easier than pollen germination tests; however, in some cases, germination tests are essential to examine the actual viability of pollen [41].

In this study, TTC staining, together with in vitro germination tests, was conducted to assess the pollen viability of *P.* Queen Beer ‘Mantefon’. Both tests revealed pollen sterility, in which the orchid pollinia remained unstained, and no pollen tube growth was observed in any of the 10 flower developmental stages examined. The pollinia of flower buds, with sizes ranging from 13 to 20 mm, were evaluated using TTC staining and an in vitro pollen germination test. Although the pollinia of all flower buds showed heavy TTC staining, the pollen tubes failed to grow, resulting in a germination rate of 0%. The TTC test relies on the reduction in colorless and water-soluble TTC to an insoluble red compound (formazan). This reduction occurs when hydrogen ions are donated to TTC upon dehydrogenase activity in metabolically active tissues [50,51]. Pollinia from the flower buds were stained with TTC, probably since they were metabolically active. However, these pollinia were still in the early stages of meiosis and had not yet formed microspores; therefore, the pollen tubes failed to grow.

Normal meiotic processes, including proper chromosome segregation, are directly related to plant fertility. Therefore, hybrid infertility is associated with abnormal meiosis during gamete formation. Abnormalities and irregularities in meiotic pairings, such as the presence of univalent and multivalent chromosomes, are typically observed in distant orchid hybrids [19,21,52]. Triploids are often meiotically unstable, which leads to frequent chromosomal loss and fragmentation [53]. The meiotic analysis in *P.* Queen Beer ‘Mantefon’ identified aberrations in chromosome pairing and segregation. In prophase I at the pachytene stage, ‘Mantefon’ presented unpaired and partially paired regions, possibly caused by a lack of chromosome homology of these regions and reduced recombination [54]. Eccentric chromosomal configurations, including trivalents and univalents, occurred frequently during diakinesis until early metaphase I, which led to the meiotic irregularities observed in the subsequent meiotic stages. Low bivalent formation in hybrids with prominently dissimilar genomes may account for the differences in the number of repetitive sequences between the parental genomes. This discrepancy influences the structural homology of the parental chromosomes and subsequently reduces their ability to pair and recombine [55]. The prior chromosomal abnormalities presented can lead to the production of lagging chromosomes and micronuclei [56,57] as observed in ‘Mantefon’. Chromosome bridges have also been detected in this cultivar, suggesting that chromosome structural variations may have occurred [58]. Meiosis results in a high frequency of tetrads and polyads, as well as several sporad types with varying numbers of micronuclei. All types of meiotic irregularities found in *P.* Queen Beer ‘Mantefon’ might be attributed to reduced pollen sterility.

The differences in chromosome size hinder successful crossbreeding between two species or genera due to impaired chromosome pairing during meiosis [59], which were all observed in *P.* Queen Beer ‘Mantefon’. Determination of the ploidy level as well as analysis of the meiotic chromosome behaviors of significant breeding stocks, including wide species, cultivars, and parental hybrids, will enhance the breeding efficiency in *Phalaenopsis* by proper selection of the parents for hybridization based on their cytogenetic information [60].

## 4. Materials and Methods

### 4.1. Plant Materials and Sample Collection

Young, healthy roots and varied sizes of tightly closed flower buds of *P.* Queen Beer ‘Mantefon’ were harvested from the plants provided by Kangsan Orchids, Republic of Korea. Roots were treated with 2 mM 8-hydroxyquinoline for 5 h at 25 °C to arrest cells at metaphase, fixed in Carnoy’s solution (3:1 ethanol–acetic acid) overnight, and stored in 70% ethanol until use for mitotic chromosome preparation. Flower buds of different sizes were collected, fixed in the same fixative for 24 h, and then stored at 4 °C in 70% ethanol. Meiotic stages were determined according to the longitudinal bud length at the time of collection. More than 50 developing flower buds and flowers were collected.

Pollinia of *P.* ‘KS Little Gem’ at anthesis and *P.* Queen Beer ‘Mantefon’ at ten (10) flower developmental stages (Figure 2A) were harvested for viability comparison through TTC staining and in vitro germination ability. The tetraploid *P.* ‘KS Little Gem’ was used as control for the pollen viability assays. Flower development stages were defined as follows: (1) Tight flower bud, (2) sepal half-open, (3) flower half-open, (4) prior to anthesis, (5) flower fully open, (6) one day after fully open, (7) two days after fully open, (8) three days after fully open, (9) four days after fully open, and (10) five days after fully open.

### 4.2. Somatic Chromosome Preparation

Chromosome samples were prepared as described by Lim et al. [61] with minor modifications. Fixed root tips were washed with distilled water before treatment with an enzyme mixture (1% cellulose, cytohelicase, and pectolyase) at 37 °C for 90 min. Thereafter, the enzyme-treated roots were transferred to 1.5 mL tubes containing Carnoy’s solution and vortexed for 20 s. Homogenized root meristems were placed on ice for 5 min and centrifuged at 13,000 rpm. The resulting supernatant was discarded, and the pelleted material was resuspended in an acetic acid–ethanol (9:1) solution. Samples of the suspensions were dropped onto pre-warmed (80 °C) glass slides in a humid chamber and left to air dry at room temperature.

### 4.3. Meiotic Chromosome Preparation

Chromosome spreads were prepared as described by Park et al. [62] with minor modifications. The fixed buds were rinsed with distilled water. The pollinia were removed and macerated with 1% cellulose, cytohelicase, and pectolyase in 10 mM citrate buffer at 37 °C for 60 min. Digested samples were squashed in droplets of the same fixative. After drying, the slides were stained with 1 µg mL^−1^ 4′,6-diamidino-2-phenylindole (DAPI) in Vectashield (Vector Labs, H-1000, Burlingame, CA, USA) and examined under a fluorescence microscope (BX53, Olympus, Tokyo, Japan) with a built-in CCD camera (CoolSNAP™cf, Photometrics, Tucson, AZ, USA) using an oil lens (×100 magnification).

### 4.4. Fluorescence in Situ Hybridization

5S rDNA and 45S rDNA oligoprobes were used for FISH karyotype analysis. Pre-labeled oligoprobes were prepared using the method described by Waminal et al. [63]. FISH was performed as described by Lim et al. [64] with minor modifications. The hybridization mixture consisted of 50% formamide, 10% dextran sulfate, 20× SSC, 50 ng μL^−1^ of each DNA probe, and Sigma purified water.f The mixture was pipetted onto prepared chromosomal slides and denatured on an 80 °C slide heater for 5 min. The slides were then incubated at room temperature in a humid chamber for 30 min. After hybridization, the slides were washed successively with 2× SSC at room temperature for 10 min, 0.1× SSC at 42 °C for 25 min, and 2X SSC at room temperature for 5 min and then dehydrated through an ethanol series (70%, 90%, and 100%) at room temperature for 3 min each. The slides were counterstained with Vectashield (Vector Labs, H-1000, USA) with 1 µg DAPI and examined under an Olympus BX53 fluorescence microscope with a built-in CCD camera (CoolSNAP™cf) using an oil lens (×100 magnification). Images were enhanced using Adobe Photoshop (CS6, Adobe, San Jose, CA, USA). The chromosomes were paired based on the FISH signals, morphological characteristics, and chromosome length.

### 4.5. Sporad Quantification in Pollen Mother Cells

Pollinia from full-sized flower buds and at early anthesis were fixed in 3:1 Carnoy’s solution for 24 h, hydrolyzed in 1 N HCl for 1 h at 60 °C, rinsed three times with distilled water, and stained with DAPI. The materials used for observation were examined under a fluorescence microscope. At least 300 PMCs were categorized according to the number of sporads at the end of division II.

### 4.6. Pollen Viability by TTC Staining

Pollinia viability was assessed by staining with TTC (Sigma Aldrich, Saint Louis, MI, USA) [42,43,45]. Fresh pollinia were placed in a 1.5 mL centrifuge tube containing 0.5% TTC aqueous solution and incubated for 24 h at 25 ± 1 °C in the dark. A 0.5% TTC aqueous solution was prepared by dissolving TTC (0.5 g) in 50 mM sodium phosphate buffer solution (pH 7.4). The stained pollinia were rinsed with distilled water to remove any additional stains. The stained pollinia were examined under a stereomicroscope (SZ 51; Olympus, Tokyo, Japan). The area of pollinia stained red was considered viable, whereas the unstained pollinia portion was considered non-viable.

### 4.7. In Vitro Pollen Germination

Pollinia were dipped in 70% alcohol for surface sterilization, cultured in a germination medium containing 100 g/L sucrose, 0.02 g/L boric acid, and 5 g/L plant agar, pH 5.8, autoclaved at 121 °C for 20 min, and then incubated at room temperature in the dark for 10 days. The pollinia were squashed in the DAPI solution as evenly as possible. The pollen tube was visualized under a microscope at 600× magnification, and approximately 100 pollen grains were observed for each stage. Pollen grains were considered to have germinated when the pollen tube was elongated to twice the pollen grain size. Pollen germination levels were ranked as follows: 0, no germination; 1, 1–20%; 2, 21–40%; 3, 41–60%; 4, 61–80%; 5, 81–100% germination.

## 5. Conclusions

Mitotic and meiotic chromosome analyses, as well as pollen viability tests, provide beneficial information for selecting the best candidates with more stable genotypes for the introgression of genes from one species to another through hybridization. Pollen fertility is associated with abnormal meiosis caused by deficient pairing, non-separation, or asymmetrical chromosome distribution [65]. These suggestions are supported by our findings on the extremely abnormal chromosome behavior of the triploid cultivar, *P.* Queen Beer ‘Mantefon’ throughout the meiotic process. The results of this study provide detailed cytogenetic information and insights into the possible causes of sterility in this cultivar, which could be useful for planning effective breeding programs for *Phalaenopsis*.

## Figures and Tables

**Table 1:** Meiotic pairing configurations in *Phalaenopsis* Queen Beer ‘Mantefon’.

Chromosome No.	No. of Metaphase I Cells	Pairing Configuration		
		Univalents	Bivalents	Trivalents
57	35	7 (1–19)	20.3 (19–28)	1.3 (1–4)

**Table 2:** Frequency of abnormal meiosis in *Phalaenopsis* Queen Beer ‘Mantefon’.

Stage	Abnormality (%)	Normality (%)
Prophase I	64.93% (87/134) ^1^	35.07% (47/134) ^2^
Metaphase I	98.77% (80/81)	1.23% (1/81)
Anaphase I	83.72% (36/43)	16.28% (7/43)
Metaphase II	80% (8/10)	20% (2/10)
Anaphase II	92.6% (25/27)	7.40% (2/27)
Tetrad Stage	21.30% (69/324)	78.70% (255/324)

^1^ Number of PMCs showing abnormal meiosis/number of observed PMCs; ^2^ number of PMCs showing normal meiosis/number of observed PMCs.

**Table 3:** Distribution of sporad types observed in the staining of pollen mother cells with DAPI solution.

Total Number of Sporocytes	Pollen Mother Cells (Percentage %)						
	Monad	Dyad	Triad	Tetrad	Polyad	Tetrad with Micronucleus	Sporads with 1 Micronucleus
324	5 (2%)	6 (2%)	18 (6%)	69 (21%)	35 (11%)	141 (44%)	50 (15%)

## Data Availability

The data presented in this study are available within the article.

## References

[1] Guo W.J., Lin Y.Z., Lee N. (2012). Photosynthetic light requirements and effects of low irradiance and daylength on *Phalaenopsis amabilis*. J. Am. Soc. Hortic. Sci..

[2] Lin M.J., Hsu B.D. (2004). Photosynthetic plasticity of *Phalaenopsis* in response to different light environments. J. Plant Physiol..

[3] United States Department of Agriculture (USDA) (2018). Floriculture Crops 2017 Summary.

[4] United States Department of Agriculture (USDA) (2021). Floriculture Crops 2020 Summary.

[5] Cho A.R., Chung S.W., Kim Y.J. (2022). Shortening the vegetative growth stage of *Phalaenopsis* Queen Beer ‘Mantefon’ by controlling light with calcium ammonium nitrate levels under enriched CO_2_. Horticulturae.

[6] Lee Y.I., Tseng Y.F., Lee Y.C., Chung M.C. (2020). Chromosome constitution and nuclear DNA content of *Phalaenopsis* hybrids. Sci. Hortic..

[7] Tang C.Y., Chen W.H., Chen W.H., Chen H.H. (2007). Breeding and development of new varieties in *Phalaenopsis*. Orchid Biotechnology.

[8] Lin C., Chen Y., Chen W., Chen C., Kao Y. (2005). Genome organization and relationships of *Phalaenopsis* orchids inferred from genomic in situ hybridization. Bot. Bull. Acad. Sin..

[9] Christenson E.A. (2001). Phalaenopsis: A monograph.

[10] Singh R.J. (2003). Plant cytogenetics.

[11] Shin H., Park H.R., Park J.E., Yu S.H., Yi G., Kim J.H., Koh W., Kim H.H., Lee S.S., Huh J.H. (2021). Reduced fertility caused by meiotic defects and micronuclei formation during microsporogenesis in *xBrassicoraphanus*. Genes. Genom..

[12] Mercier R., Mézard C., Jenczewski E., Macaisne N., Grelon M. (2015). The molecular biology of meiosis in plants. Ann. Rev. Plant Biol..

[13] Gray S., Cohen P.E. (2016). Control of meiotic crossovers: From double-strand break formation to designation. Ann. Rev. Genet..

[14] Wang Y., van Rengs W.M., Zaidan M.W.A.M., Underwood C.J. (2021). Meiosis in crops: From genes to genomes. J. Exp. Bot..

[15] Boff T., Schifino-Wittmann M.T. (2002). Pollen fertility and meiotic behaviour in accessions and species of *Lleucaena*. Trop. Grass.

[16] Cifuentes M., Eber F., Lucas M.O., Lode M., Chèvre A.M., Jenczewski E. (2010). Repeated polyploidy drove different levels of crossover suppression between homoeologous chromosomes in *Brassica napus* allohaploids. Plant Cell.

[17] Szadkowski E., Eber F., Huteau V., Lode M., Huneau C., Belcram H., Coriton O., Manzanares-Dauleux M.J., Delourme R., King G.J. (2010). The first meiosis of resynthesized *Brassica napus*, a genome blender. New Phytol..

[18] Lee Y.H. (1987). Cytology and fertility of an intergeneric orchid hybrid. J. Hered..

[19] Kamemoto H., Amore T.D., Kuehnle A.R. (1999). Breeding Dendrobium Orchids in Hawaii.

[20] Bolanos-Villegas P., Chin S.W., Chen F.C. (2008). Meiotic chromosome behavior and capsule setting in Doritaenopsis hybrids. J. Am. Soc. Hortic. Sci..

[21] Lee Y.I., Chang F.C., Chung M.C. (2011). Chromosome pairing affinities in interspecific hybrids reflect phylogenetic distances among lady’s slipper orchids (*Paphiopedilum*). Ann. Bot..

[22] Kim C., Robertson J.S., Paterson A.H. (2011). Inference of subgenomic origin of BACs in an interspecific hybrid sugarcane cultivar by overlapping oligonucleotide hybridizations. Genome.

[23] Li C., Dong N., Zhao Y., Wu S., Liu Z. (2021). A review for the breeding of orchids: Current achievements and prospects. Hortic. Plant J..

[24] Kopecký D., Martis M., Číhalíková J., Hřibová E., Vrána J., Barto¡ J., Kopecká J., Cattonaro F., Stočes Š., Novák P. (2013). Flow sorting and sequencing meadow fescue chromosome 4F. Plant Physiol..

[25] Harper J., Armstead I., Thomas A., James C., Gasior D., Bisaga M., Roberts L., King I., King J. (2011). Alien introgression in the grasses *Lolium perenne* (perennial ryegrass) and *Festuca pratensis* (meadow fescue): The development of seven monosomic substitution lines and their molecular and cytological characterization. Ann. Bot..

[26] Moscone E.A., Matzke M.A., Matzke A.J.M. (1996). The use of combined FISH/GISH in conjunction with DAPI counterstaining to identify chromosomes containing transgene inserts in amphidiploid tobacco. Chromosoma.

[27] Jacobsen E., De Jong J.H., Kamstra S.A., Van den Berg P.M., Ramanna M.S. (1995). Genomic in situ hybridization (GISH) and RFLP analysis for the identification of alien chromosomes in the backcross progeny of potato (+) tomato fusion hybrids. Heredity.

[28] Palma-Silva C., dos Santos D.G., Kaltchuk-Santos E., Bodanese-Zanettini M.H. (2004). Chromosome numbers, meiotic behavior, and pollen viability of species of *Vriesea* and *Aechmea* genera (Bromeliaceae) native to Rio Grande do Sul, Brazil. Am. J. Bot..

[29] Filippi M., Boldrini K.R., Agostinho K.F., Corrêa B.J.S., Donazzolo J. (2022). Cytogenetic study and pollen viability of *Diatenopteryx sorbifolia* Radlk. Ciênc. Florest..

[30] Griesbach R.J. (1985). Polypioidy in *Phalaenopsis* orchid improvement. J. Hered..

[31] Lim K.B., Wennekes J., Jong J.H.D., Jacobsen E., Van Tuyl J.M. (2001). Karyotype analysis of *Lilium longiflorum* and *Lilium rubellum* by chromosome banding and fluorescence in situ hybridisation. Genome.

[32] Maluszynska J., Heslop-Harrison J.S. (1993). Physical mapping of rDNA loci in *Brassica* species. Genome.

[33] Maluszynska J., Heslop-Harrison J.S. (1993). Molecular cytogenetics of the genus *Arabidopsis*: In situ localization of rDNA sites, chromosome numbers and diversity in centromeric heterochromatin. Ann. Bot..

[34] Galasso I., Schmidt T., Pignone D., Heslop-Harrison J.S. (1995). The molecular cytogenetics of *Vigna unguiculata* (L.) Walp: The physical organization and characterization of 18 s-5.8 s-25 s rRNA genes, 5 s rRNA genes, telomere-like sequences, and a family of centromeric repetitive DNA sequences. Theor. Appl. Genet..

[35] Pacini E. (1997). Tapetum character states: Analytical keys for tapetum types and activities. Can. J. Bot..

[36] Pacini E. (2009). Orchid pollen dispersal units and reproductive consequences. Orchid. Biol. Rev. Perspect..

[37] Pacini E., Hesse M. (2002). Types of pollen dispersal units in orchids, and their consequences for germination and fertilization. Ann. Bot..

[38] Souza M.D., Pereira T.N.S., Martins E.R. (2002). Microsporogenesis and microgametogenesis associated with flower bud and anther size and pollen viability in yellow passion fruit (*Passiflora edulis* Sims f. flavicarpa Degener). Sci. Agrotechnol.

[39] Ottaviano E., Mulcahy D.L. (1989). Genetics of angiosperm pollen. Adv. Genet..

[40] Shivanna K.R., Ram H.M. (1993). Pollination biology: Contributions to fundamental and applied aspects. Curr. Sci..

[41] Sulusoglu M., Cavusoglu A. (2014). In vitro pollen viability and pollen germination in cherry laurel (*Prunus laurocerasus* L.). Sci. World J..

[42] Mattson A.M., Jensen C.O., Dutcher R.A. (1947). Triphenyltetrazolium chloride as a dye for vital tissues. Science.

[43] Khatun S., Flowers T.J. (1995). The estimation of pollen viability in rice. J. Exp. Bot..

[44] Sorkheh K., Shiran B., Rouhi V., Khodambashi M. (2011). Influence of temperature on the in vitro pollen germination and pollen tube growth of various native Iranian almonds (*Prunus* L. spp.) species. Trees.

[45] Abdelgadir H.A., Johnson S.D., Van Staden J. (2012). Pollen viability, pollen germination and pollen tube growth in the biofuel seed crop *Jatropha curcas* (Euphorbiaceae). S. Afr. J. Bot..

[46] Alexander M.P. (1969). Differential staining of aborted and nonaborted pollen. Stain. Technol..

[47] Heslop-Harrison J., Heslop-Harrison Y. (1970). Evaluation of pollen viability by enzymatically induced fluorescence; intracellular hydrolysis of fluorescein diacetate. Stain. Technol..

[48] Van Der Walt I.D., Littlejohn G.M. (1996). Storage and viability testing of *Protea* pollen. J. Am. Soc. Hortic. Sci..

[49] Bellusci F., Musacchio A., Stabile R., Pellegrino G. (2010). Differences in pollen viability in relation to different deceptive pollination strategies in Mediterranean orchids. Ann. Bot..

[50] Junillon T., Morand L., Flandrois J.P. (2014). Enhanced tetrazolium violet reduction of *Salmonella* spp. by magnesium addition to the culture media. Food Microbiol..

[51] Lopez Del Egido L., Navarro-Miró D., Martinez-Heredia V., Toorop P.E., Iannetta P.P. (2017). A spectrophotometric assay for robust viability testing of seed batches using 2, 3, 5-triphenyl tetrazolium chloride: Using *Hordeum vulgare* L. as a model. Front. Plant Sci..

[52] Arends J.C. (1970). Cytological observations on genome homology in eight interspecific hybrids of *Phalaenopsis*. Genetica.

[53] McClintock B. (1929). Chromosome morphology in *Zea mays*. Science.

[54] Conceição S.I., Róis A.S., Caperta A.D. (2019). Nonreduction via meiotic restitution and pollen heterogeneity may explain residual male fertility in triploid marine halophyte *Limonium algarvense* (Plumbaginaceae). Caryologia.

[55] Kao Y.Y., Chang S.B., Lin T.Y., Hsieh C.H., Chen Y.H., Chen W.H., Chen C.C. (2001). Differential accumulation of heterochromatin as a cause for karyotype variation in *Phalaenopsis* orchids. Ann. Bot..

[56] Del Bosco S.F., Tusa N., Conicella C. (1999). Microsporogenesis in a Citrus interspecific tetraploid somatic hybrid and its fusion parents. Heredity.

[57] Tel-Zur N., Abbo S., Mizrahi Y. (2005). Cytogenetics of semi-fertile triploid and aneuploid intergeneric vine cacti hybrids. J. Hered..

[58] Wang J., Huo B., Liu W., Li D., Liao L. (2017). Abnormal meiosis in an intersectional allotriploid of *Populus* L. and segregation of ploidy levels in 2x× 3x progeny. PLoS ONE.

[59] Hsu C.C., Chen H.H., Chen W.H. (2018). Phalaenopsis. Ornamental Crops.

[60] Chen W.H., Tang C.Y., Kao Y.L. (2010). Polyploidy and variety improvement of *Phalaenopsis* orchids. I Int. Orchid. Symp..

[61] Lim K.B., De Jong H., Yang T.J., Park J.Y., Kwon S.J., Kim J.S., Lim M.H., Kim J.A., Jin M., Jin Y.M. (2005). 2005. Characterization of rDNAs and tandem repeats in the heterochromatin of *Brassica rapa*. Mol. Cells.

[62] Park H.R., Park J.E., Kim J.H., Shin H., Yu S.H., Son S., Yi G., Lee S.S., Kim H.H., Huh J.H. (2020). Meiotic chromosome stability and suppression of crossover between non-homologous chromosomes in x *Brassicoraphanus*, an intergeneric allotetraploid derived from a cross between *Brassica rapa* and *Raphanus sativus*. Front. Plant Sci..

[63] Waminal N.E., Pellerin R.J., Kim N.S., Jayakodi M., Park J.Y., Yang T.J., Kim H.H. (2018). Rapid and efficient FISH using pre-labeled oligomer probes. Sci. Rep..

[64] Lim K.B., Yang T.J., Hwang Y.J., Kim J.S., Park J.Y., Kwon S.J., Kim J., Choi B.S., Lim M.H., Jin M. (2007). Characterization of the centromere and peri-centromere retrotransposons in *Brassica rapa* and their distribution in related *Brassica* species. Plant J..

[65] Singh R.N. (1992). Chromosomal abnormalities and fertility in induced autotetraploid *Helianthus annuus* in the C1 and C2 generations. Cytologia.

